# Are Uropathogenic Bacteria Living in Multispecies Biofilm Susceptible to Active Plant Ingredient—Asiatic Acid?

**DOI:** 10.3390/biom11121754

**Published:** 2021-11-24

**Authors:** Zuzanna Sycz, Dorota Tichaczek-Goska, Anna Jezierska-Domaradzka, Dorota Wojnicz

**Affiliations:** 1 Department of Biology and Medical Parasitology, Wroclaw Medical University, 50-345 Wroclaw, Poland; sycz.zuzanna@gmail.com (Z.S.); dorota.wojnicz@umw.edu.pl (D.W.); 2 Department of Pharmaceutical Biology and Botany, Wroclaw Medical University, 50-556 Wroclaw, Poland; anna.jezierska-domaradzka@umw.edu.pl

**Keywords:** asiatic acid, plant compounds, uropathogenic bacteria, urinary tract infection (UTI), multispecies biofilm

## Abstract

Urinary tract infections (UTIs) are a serious health problem in the human population due to their chronic and recurrent nature. Bacteria causing UTIs form multispecies biofilms being resistant to the activity of the conventionally used antibiotics. Therefore, compounds of plant origin are currently being searched for, which could constitute an alternative strategy to antibiotic therapy. Our study aimed to determine the activity of asiatic acid (AA) against biofilms formed by uropathogenic *Escherichia coli*, *Enterobacter cloacae*, and *Pseudomonas aeruginosa*. The influence of AA on the survival, biofilm mass formation by bacteria living in mono-, dual-, and triple-species consortia as well as the metabolic activity and bacterial cell morphology were determined. The spectrophotometric methods were used for biofilm mass synthesis and metabolic activity determination. The survival of bacteria was established using the serial dilution assay. The decrease in survival and a weakening of the ability to create biofilms, both single and multi-species, as well as changes in the morphology of bacterial cells were noticed. As AA works best against young biofilms, the use of AA-containing formulations, especially during the initial stages of infection, seems to be reasonable. However, there is a need for further research concerning AA especially regarding its antibacterial mechanisms of action.

## 1. Introduction

It is well known that biofilms formed by both commensal and pathogenic bacteria usually do not constitute a mono-species consortium but form multispecies communities of microorganisms. An example of such a multispecies biofilm is the biofilm formed in the urinary tract during its infections. The main cause of urinary tract infections (UTIs) is uropathogenic *Escherichia coli*, accompanied by other species, including *Enterobacter cloacae* and *Pseudomonas aeruginosa*. Prevention of the formation of such a mixed-species biofilm or its eradication using standard pharmacotherapy is problematic and often associated with failure. Therefore, compounds of plant origin are currently being searched for, which could constitute an alternative strategy to antibiotic therapy or support it in preventing the formation of bacterial biofilm or facilitate its removal. 

**Figure 1:**
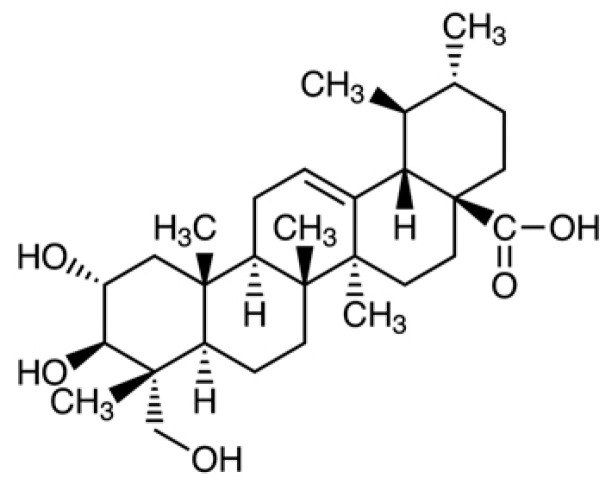
Chemical formula of asiatic acid.

Asiatic acid (AA; 2α,23-dihydroxyursolic acid) belongs to the pentacyclic triterpenes group, polycyclic compounds classified as secondary plant metabolites. It is a monocarboxylic acid derived from the hydride of a ursane. In the structural formula, ursane is substituted with a carboxyl group at C-28 and hydroxyl groups at C-2, C-3, and C-23 (stereoisomer 2α, 3β) (Figure 1). The molecular formula can be presented as C30H48O5, the molecular weight of AA is 488.70 g/mol [1,2]. AA can be found in the bark, cork cambium, resin, epidermis, waxy coating on leaves, and flowers of many plant species, where it protects against insect and microbial attack.

In the form of asiaticoside and aglycone, large amounts of AA are found in the herb of *Centella asiatica* (L.) Urb. (Apiaceae), also known as “gotu kola” and “tiger herb”. The total fraction of TPs of *C. asiatica* contains 30% AA, 40% asiaticoside and 30% madecassic acid. *C. asiatica* can be found in South Africa, Australia, Oceania, and Southeast Asian countries (mainly India and China, but also Japan, Malaysia, and Indonesia). It is used both in the traditional far eastern and modern western phytotherapy due to the wide range of valuable pro-health properties and supporting the treatment of many diseases, especially those with inflammation [3,4,5,6]. The in vitro and in vivo studies so far have confirmed a number of pharmacological properties of AA, which include anti-cancer, hypotensive, cardioprotective, anti-infarction, anti-stroke, antihyperlipidemic, antidiabetic, hepatoprotective, gastroprotective, nephroprotective, diuretic, immunostimulatory, neuroprotective, nootropic, anti-Parkinson’s, anti-Alzheimer’s, anti-osteoporosis, antimalarial, antifungal, antiviral, anti-photoaging, supporting the treatment of burns and non-healing diabetic wounds, as well as a spermicidal effect [1,2,7,8,9,10]. Nowadays, in view of the fact that this plant shows an overall health-promoting effect, in its countries of origin it is consumed prophylactically both in the form of nutraceutical preparations and as an addition to salads and drinks. Moreover, *C. asiatica* is an ingredient in ointments, cosmetics, and toothpaste [11,12].

However, few literature reports describe the antimicrobial properties of AA. The experiments carried out in recent years have proved that this acid has a significant antibacterial effect when used alone as well as in combination with antibiotics. These studies focused primarily on bacteria living in suspension, i.e., planktonic forms. They included the determination of the MIC value (minimum inhibitory concentration) of the acid, its effect on bacterial survival, cell morphology and integrity of the bacterial membrane, and the impact on bacterial virulence factors such as hydrophobicity of the cell’s surface, ability to move, synthesis of curli fimbriae and P-type fimbriae [13,14,15,16,17,18,19,20,21,22]. Up to date, few studies have investigated the effect of AA on the ability of bacteria to form biofilms, and these studies have mainly concentrated on single-species consortia [21,22,23,24]. 

The impact of AA on multispecies biofilms has not been described so far. Therefore, our research aimed to determine the effect of AA on the growth dynamics and the number of uropathogenic bacteria living in biofilms. The amount of biofilm mass formed and its structure, cell morphology, and metabolic activity of bacilli living in mono- (*E. coli*; *E. cloacae*; *P. aeruginosa*), dual- (*E. coli + E. cloacae*; *E. coli + P. aeruginosa*), and triple-species biofilms (*E. coli + E. cloacae + P. aeruginosa*) at various stages of their development (adhesion, maturation, dispersion, and migration) were also established.

## 2. Materials and Methods

### 2.1. Bacterial Strains

Three uropathogenic reference strains from the American Type Culture Collection (ATCC) were used in the study: *Escherichia coli* CFT073, *Enterobacter cloacae* ATCC-BAA 2468, *Pseudomonas aeruginosa* ATCC 25000.

### 2.2. Asiatic Acid

Asiatic acid (AA, purity 97%) was purchased from Sigma-Aldrich (Poland). Stock AA with a concentration of 4 mg/mL was prepared each time by dissolving the acid in an aqueous DMSO solution. For further experiments, stock AA was diluted with the appropriate culture medium.

### 2.3. MIC (Minimal Inhibitory Concentration) and MBC (Minimal Bactericidal Concentration) Determination

The MIC and MBC values of AA against examined bacterial strains were established using the 2-fold microdilution method according to CLSI [25]. The MIC was the lowest concentration of AA that prevented the visible growth of bacteria in the microtitre plate. The MBC was the lowest concentration of AA that killed bacteria. The MBC value was determined by subculturing bacterial samples to agar plates.

### 2.4. Preparation of the Bacterial Suspension

Strains cultures were grown overnight on TSB (Tripticasein Soy Broth, Biocorp, Poland) at 37 °C. Then, bacteria were transferred to fresh TSB and incubated at 37 °C for 2 h in the shaking water bath (Julabo SW-22, Poland). Following incubation, while bacterial cells were in the log phase of growth, the bacterial suspensions were centrifuged and suspended in PBS to reach the final density of 0.5 McFarland (10^8^ CFU/mL). The suspensions prepared in this way were used in the experiments.

### 2.5. Biofilm Cultures

Cultures were carried out in 96-well polystyrene microtiter plates for 6, 24, 48, 72, and 96 h. Single species (*E. coli, E. cloacae, P. aeruginosa*), dual-species (*E. coli + E. cloacae*; *E. coli + P. aeruginosa*), and triple-species (*E. coli + E. cloacae + P. aeruginosa*). The control biofilm samples did not contain AA, while the test samples contained AA at a concentration of 0.5 × MIC.

### 2.6. The Bacterial Cell Count in Biofilm Cultures

Bacterial survival in biofilm was established after each time of incubation: 6, 24, 48, 72, and 96 h. Biofilm cultures were gently washed to remove cells unattached to the biofilm matrix. The biofilm deposited on the walls of the microtiter plate wells was scraped by hand with a sterile spatula, transferred to microtubes containing PBS, and vortexed for 3 min to disperse the biofilm uniformly [26]. The CFU/mL values were assessed by plating serial dilutions on chromogenic coliform agar (Graso Biotech, Poland).

### 2.7. Determination of the Biofilm Mass by Spectrophotometric Method

Unbound cells were removed from the 6, 24, 48, 72, and 96 h biofilm cultures by gentle washing three times with sterile PBS. Then, 1% crystal violet (CV) was added to each well and incubated at 37 °C for 15 min in order to penetrate the CV into the structure of the biofilm. The dye was then removed, and 95% ethanol was added to elute the CV from the biofilm matrix. After 15-min incubation at room temperature, the optical density (OD) was measured at a wavelength of 590 nm on a microplate reader (HiPo MPP-96^®^ BIOSAN, Poland). On the basis of the OD value, the bacteria were classified into one of the following groups: OD ≤ ODc—non-biofilm producer; ODc < OD ≤ 2 × ODc—weak-biofilm producer; 2 × ODc < OD ≤ 4 × ODc—moderate-biofilm producer; 4 × ODc < OD—strong-biofilm producer. The ODc value was calculated as the sum of the mean OD values for the blank (TSB) and 3 times the standard deviation of the mean OD value for the TSB [27,28,29]. The ODc value was 0.218.

### 2.8. Determination of the Metabolic Activity of Bacteria in a Biofilm Using the Spectrophotometric Method

2,3,5-triphenyl tetrazolium chloride (TTC) (10%) was added to 6, 24, 48, 72, and 96 h biofilm cultures. The metabolically active bacteria reduced TTC to red triphenyl formazan. After 24 h of incubation at 37 °C, plankton cells were removed by washing the biofilm cultures with sterile PBS. Then, 95% ethanol was added to each culture and after 15 min of incubation at room temperature, the amount of produced triphenyl formazan was measured spectrophotometrically at a wavelength of 490 nm (HiPo MPP-96^®^ BIOSAN, Poland) [30,31].

### 2.9. Effect of AA on Bacterial Cell Morphology

Bacterial biofilms were incubated at 37 °C for 6, 24, 48, 72, and 96 h with AA at a concentration of 0.5 × MIC. Washed with PBS samples were then Gram-stained and observed under the microscope (Nikon Eclipse 400). The changes in bacterial cell morphology were recorded.

### 2.10. Statistical Analysis

Each experiment was repeated three times (so-called technical repeat); the grown bacterial colonies were counted from 6–8 plates, and the optical density was read from 6 wells of the microtiter plate, which gives a total of 18–24 so-called biological repeats. The final results are average values. The nonparametric Kruskal–Wallis test followed by a Dunn’s multiple comparison test were used to analysis of the obtained results. Statistical calculations were made using Statistica 13.3. (Stat Soft, Kraków, Poland). Values of *p* ≤ 0.05 were considered statistically significant.

## 3. Results and Discussion

### 3.1. MICs and MBCs Determination

The susceptibility of bacterial strains to analyzed plant compounds is specified by determining the MIC and MBC values. The MIC and MBC values of AA against the Gram-negative reference strains used in this study were 1536 and 2048 μg/mL for both *E. coli* CFT073 and *P. aeruginosa* ATCC25000, as well as 1024 and 1536 μg/mL for *E. cloacae* ATCC-BAA2468, respectively (Table 1). 

In our previous study concerning the antimicrobial activity of AA against clinical *E. coli* strains, it was found that the MIC values of AA were also high, ranging from 512 to >1024 μg/mL [17,18]. The MIC values of AA against *P. aeruginosa* PAO1, *P. aeruginosa* ATCC 27853, and *E. coli* ATCC2 5922 obtained by Garo et al. [23] and Acebey-Castellon et al. [14] were >128 μg/mL. There were significantly lower MIC and MBC values of AA against *E. coli* O157:H7, *Salmonella typhimurium* DT104, and *P. aeruginosa* and they were 20–40 μg/mL and 32–46 μg/mL, respectively [19]. The differences of MICs of AA for bacteria belonging to the same species may be due to the different origins (sources). Strains used in the current study are classified as uropathogenic. According to the American Type Culture Collection (ATCC), *E. coli* CFT073 has been isolated from blood and urine from a woman with acute pyelonephritis and *P. aeruginosa* ATCC 2500—from the urine of a catheterized patient. Garo et al. [23] used *P. aeruginosa* PAO1 from an infected wound; Acebey-Castellon et al. [14] used *E. coli* ATCC 25922 clinical isolate of unknown origin and *P. aeruginosa* ATCC 27853 isolated from blood culture. Liu et al. [19] tested enterohemorrhagic *E. coli* O157:H7 being a major foodborne pathogen and *P. aeruginosa* isolated from products of animal origin. Moreover, the differences in MICs of AA obtained by us and those of other researchers may be caused by different methods used to determine them. In our study the microdilution method was used, other researchers used the disc diffusion method. 

Several studies have also determined the effect of AA on Gram-positive strains. As a rule, MIC and MBC values of AA against strains that belong to this group are lower compared to Gram-negative strains. Liu et al. [19] determined the susceptibility of *Listeria monocytogenes*, *Staphylococcus aureus*, *Enterococcus faecalis*, and *Bacillus cereus* to AA. The MIC and MBC values ranged between 18–44 μg/mL and 28–56 μg/mL, respectively. Even lower MIC values of AA, 10–20 μg/mL, were obtained by Harnvoravongchai et al. [22] against *Clostridium difficile* strains. However, it should be noted that the MIC values of AA against Gram-positive strains are not always as low as in above-mentioned studies. According to our other previous studies [21], those values against clinical *E. faecalis* strains were 64–128 μg/mL. Comparable results were obtained by Acebey-Castellon et al. [14] against *S. aureus* ATCC25923 (MIC > 128 μg/mL) and *E. faecalis* ATCC29212 (MIC = 128 μg/mL). According to studies conducted on *S. aureus* strains, the MIC and MBC values of AA ranged between 20–160 μg/mL [32].

When comparing the antibacterial activity of different compounds against Gram-negative and Gram-positive strains, it can be observed that the MIC values are generally higher against the former. This is related to differences in terms of the structure of cell envelopes of these two groups of bacteria. The outer membrane is a significant structure found in Gram-negative bacteria, which has a specific chemical structure that impedes the penetration of antibacterial compounds into the cell. It should be noted that bacteria that belong to the same group (Gram-positive/Gram-negative), or even to the same species, may differ significantly in their susceptibility to the same antibacterial compounds, resulting in different MIC values. The reason for these differences should also be found in various sources of microbes (clinical, environmental, or reference strains) and their individual characteristics.

### 3.2. The E. coli Cell Count in Mono-, Dual-, and Triple-Species Biofilms Untreated with AA

**Figure 2:**
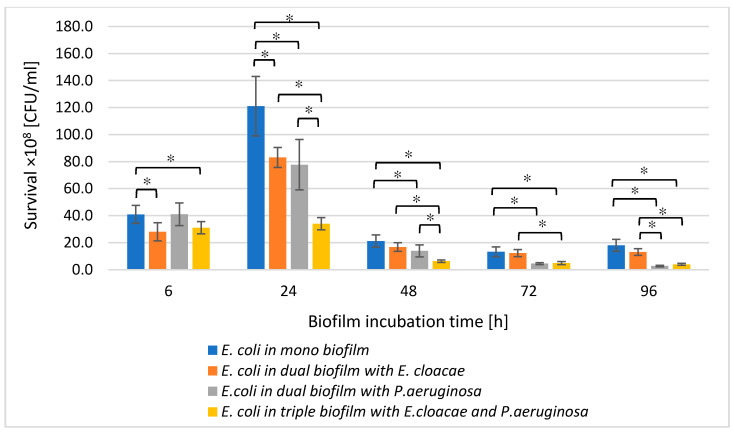
The survival of *E. coli* rods in mono-, dual-, and triple-species biofilms. Statistically significant differences were noted with an asterisk (* *p* ≤ 0.05).

Comparing the number of live *E. coli* cells in mono-species biofilm and dual-species consortia (*E. coli + E. cloacae* and *E. coli + P. aeruginosa*), it was found that the presence of either *E. cloacae* or *P. aeruginosa* in the bacterial cultures limited the growth of *E. coli* rods (Figure 2).

This may indicate antagonistic interactions between bacteria. However, statistically significant inhibition of *E. coli* growth (*p* ≤ 0.05) was noted in the *E. coli + E. cloacae* biofilm only in 6 and 24 h cultures. When comparing the viable *E. coli* cells count in the mono-species biofilm and in the dual-species consortium co-created with *P. aeruginosa*, it was found that the presence of *P. aeruginosa* significantly limited the growth of *E. coli* rods at all stages of biofilm formation (*p* ≤ 0.05) except for the young 6 h culture. The data contained in Figure 2 also show that the simultaneous presence of *E. cloacae* and *P. aeruginosa* in the triple-species biofilm (*E. coli + E. cloacae + P. aeruginosa*) significantly limited the *E. coli* growth at all stages of its formation compared to the number of *E. coli* cells growing in mono-species biofilm as well as dual-species biofilm co-created with *E. cloacae* (*p* ≤ 0.05). The reduction in *E. coli* growth in the triple-species consortium compared to the number of these rods in the dual-species biofilm co-created with *P. aeruginosa* was noticed only in 24 and 48 h samples (*p* ≤ 0.05).

### 3.3. The E. cloacae Cell Count in Mono-, Dual- and Triple-Species Biofilms Untreated with AA

**Figure 3:**
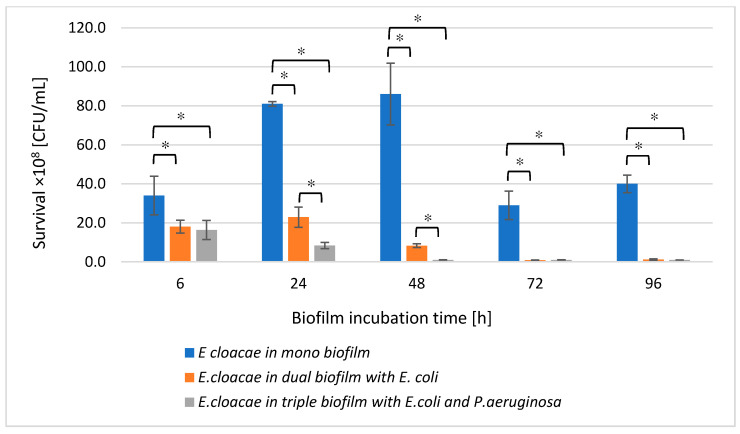
The survival of *E. cloacae* rods in mono-, dual-, and triple-species biofilms. Statistically significant differences were noted with an asterisk (* *p* ≤ 0.05).

Comparing the number of living *E. cloacae* cells in a single-species biofilm with their number in dual-species (*E. coli + E. cloacae*) and triple-species (*E. cloacae + E. coli + P. aeruginosa*) biofilms, a statistically significant reduction in *E. cloacae* cells count was found in both multispecies biofilms at all stages of their development (*p* ≤ 0.05). Such a result may be due to the antagonistic interactions between bacteria (Figure 3). Statistically significant differences in the number of *E. cloacae* cells were noted in 24 and 48 h dual-species biofilms compared to triple-species biofilms (*p* ≤ 0.05).

### 3.4. The P. aeruginosa Cell Count in Mono-, Dual- and Triple-Species Biofilms Untreated with AA

**Figure 4:**
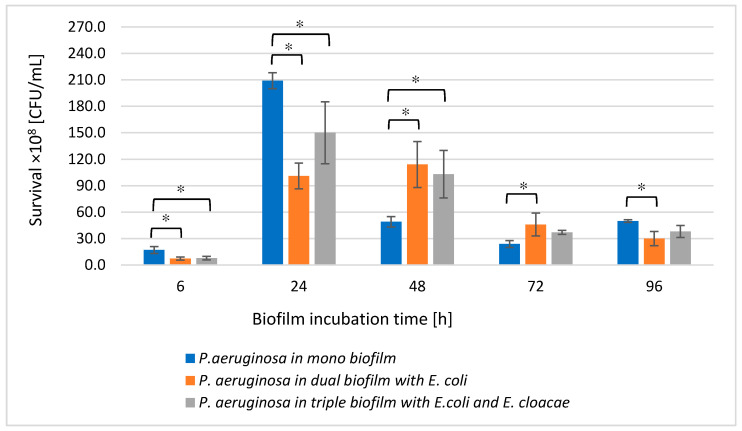
The survival of *P. aeruginosa* rods in mono-, dual-, and triple-species biofilms. Statistically significant differences were noted with an asterisk (* *p* ≤ 0.05).

As shown in Figure 4, the number of live *P. aeruginosa* cells in the dual-species biofilm (*P. aeruginosa* + *E. coli*) compared to the monoculture was significantly lower in the young 6 and 24 h biofilms and the oldest 96 h culture (*p* ≤ 0.05). In contrast, the *P. aeruginosa* cells count in the 48 and 72 h dual-species biofilms significantly increased in comparison with the number of these cells noticed in the monoculture (*p* ≤ 0.05). 

A very similar relationship was observed between single- and triple-species biofilms. Interestingly, the presence of the other bacterial species did not disturb the growth of *P. aeruginosa*, on the contrary, the *P. aeruginosa* rods grew better in mixed consortia than in the monoculture (Figure 4). 

The results obtained concerning bacterial survival indicate that single-species biofilms have higher cell counts for individual bacterial species than dual- and triple-species biofilms. This indicates the presence of mutually antagonistic interactions between microbial strains living in mixed biofilms. The growth of *E. coli* in dual-species consortia was limited by the presence of *E. cloacae* and *P. aeruginosa*; *P. aeruginosa* showed stronger antagonism to *E. coli*. It should be noted that *E. coli* cell counts were lowest when the bacilli grew in a triple-species biofilm. Similarly, the presence of *E. coli* significantly reduced the growth of *E. cloacae.* In contrast, *P. aeruginosa* growth was limited by the presence of *E. coli* only in young biofilms. The antagonism noted between *P. aeruginosa* and *E. coli* is reflected in observations made by other researchers. Machado et al. [33] analyzed 6-day single- and dual-species biofilms composed of *P. aeruginosa* ATCC10145 and *E. coli* K12MG1655. In single-species biofilms, the number of cells for each strain was higher compared to the dual-species consortium. According to Vanysacker et al. [34], biofilms formed on microfiltration membrane surfaces by *E. coli* LMG 2092T and *P. aeruginosa* PA14, the number of cells for each strain was higher in single-species consortia compared to the dual-species biofilm. Based on the above-mentioned results, it can be concluded that *E. coli* and *P. aeruginosa* interact antagonistically. Cerqueira et al. [35] obtained slightly different results. They observed that *E. coli* cell counts in single-species biofilms were higher and *P. aeruginosa* cell counts were lower than when these strains formed a common dual-species consortium. *P. aeruginosa,* however, outnumbered *E. coli* only after 48 h of collective growth. Kuznetsova et al. [36] evaluated the survival of the *E. coli* strain and different strains of *P. aeruginosa* in monocultures and in mixed dual-species cultures. Three strains of *P. aeruginosa* were used in that study: reference strain ATCC 27853, clinical strain BALG (strong-biofilm producer), and clinical strain 9–3 (weak-biofilm producer). *E. coli* cell counts decreased only in 6 h mixed biofilms formed with both clinical strains of *P. aeruginosa*. However, *E. coli* cell counts were the same in the presence of the *P. aeruginosa* reference strain. The survival rate of the ATCC 27853 strain also remained unchanged in the presence of *E. coli*. In contrast, there was also a decrease in the number of the other two clinical strains of *P. aeruginosa* in dual-species consortia; as early as 6 h of incubation for the strain that was a weak-biofilm producer and as late as 12 h for the strain that was a strong biofilm producer. Two other research teams observed a lack of antagonistic relationships in dual-species biofilms formed by *E. coli* and *P. aeruginosa*. Oliveira et al. [37] noted that in single-species biofilms formed on polystyrene surfaces by *E. coli* CECT434 and *P. aeruginosa* PAO1, the number of cells for each strain was lower compared to dual-species biofilms. Solis-Velasquez et al. [38] also showed that *E. coli* ATCC 11229 and *P. aeruginosa* ATCC 15442 cell counts in the dual-species biofilm were higher than cell counts for individual strains growing in monocultures. 

As is well known, the phenomenon of “*quorum sensing*” (QS) is one of the main factors that are significant during biofilm formation. QS helps bacteria not only regulate the number of cells within their own species, but also influence the number of bacteria of another species living in the same consortium. Mirani et al. [39] observed that in a common mixed culture of *P. aeruginosa* and *E. coli*, the number of cells for each species varied according to the stage of biofilm formation. In a young, 24 h consortium, *E. coli* was numerically superior to *P. aeruginosa.* However, the situation was reversed in the case of an older, 48 h biofilm. Only *P. aeruginosa* strains producing cis-2-decenoic acid (CDA) had antagonistic effects on *E. coli*. CDA belongs to a group of diffusible molecules that are involved in interspecies signaling and they modulate the behavior of other microbes. Interestingly, CDA molecules also had the ability to disperse the formed *E. coli* biofilm. Additionally, it was found that *P. aeruginosa* produced more CDA when cultured in a biofilm mixed with *E. coli* [40]. Rahmani-Badi et al. [41] observed that pure CDA was also capable of inhibiting the production of single- and dual-species biofilms formed by *E. coli* ATCC 25922 and *K. pneumoniae* ATCC 700603. The pure CDA also caused the dispersion of biofilms already formed by these bacterial species. In addition to CDA production, *P. aeruginosa* secretes significant amounts of extracellular rhamnolipids (RHL) and signaling molecules, including N-acyl-L-homoserine lactone (AHL) involved in QS. *P. aeruginosa* bacilli living in mixed biofilms use RHL to disperse the biofilm matrix, facilitating their access to iron and oxygen, for which they compete with other microbes. With AHL molecules activating *P.* *aeruginosa* bacilli to intense divisions, this species numerically dominates in mixed biofilms [40,42]. Cao et al. [43] found that *P. aeruginosa* PAO1C growing as colonies secreted signalling molecules of alkyl quinolones (AQ) in response to the presence of *E. coli* DH5α colonies. Interestingly, the appearance of AQ at the periphery of *E. coli* colonies occurred well before the physical fusion of colonies of these two bacterial species. It is clear that varied species of bacteria living together interact with each other. As indicated by Bhattacharjee et al. [44], more intense growth of *P. aeruginosa* in mixed biofilms formed together with *E. coli* is associated not only with QS but also with the topography of a surface on which the biofilm grows. The prevalence of *P. aeruginosa* occurs only when the culture is cultivated on a flat surface. During biofilm growth on undulating surfaces, *E. coli* bacilli are less likely to disperse. This phenomenon is related to the production of indole by *E. coli* cells, which reduces the dispersive response of these bacilli to signalling compounds secreted by *P. aeruginosa*. Moreover, indole was found to play a significant role in the survival of *E. coli* in a mixed biofilm by inhibiting the production of pyocyanin and other AHL-regulated virulence factors of *P. aeruginosa* [45].

The importance of extracellular metabolites in the interactions between *P. aeruginosa* and *E. coli* is supported by Lopes et al.’s study [46]. The authors determined the effect of supernatants containing metabolites of planktonic forms and biofilm forms of *P. aeruginosa* ATCC 10145 on the growth of both planktonic forms and biofilm forms of *E. coli* K12MG1655 and vice versa. It was found that the supernatant extracted from planktonic forms of *E. coli* stimulated biofilm formation by *P. aeruginosa* bacilli, however, it did not affect their survival. In contrast, the supernatant obtained from the planktonic culture of *P. aeruginosa* did not affect either biofilm production by *E. coli* or the survival of these bacilli. Lopes et al. [46] also showed that metabolites found in the *aeruginosa* bacilli to intense divisions, this species numerically dominates in mixed biofilms [40,42]. Cao et al. [43] found that *P. aeruginosa* PAO1C growing as colonies secreted signaling molecules of alkyl quinolones (AQ) in response to the presence of *E. coli* DH5α colonies. Interestingly, the appearance of AQ at the periphery of *E. coli* colonies occurred well before the physical fusion of colonies of these two bacterial species. It is clear that varied species of bacteria living together interact with each other. As indicated by Bhattacharjee et al. [44], more intense growth of *P. aeruginosa* in mixed biofilms formed together with *E. coli* is associated not only with QS but also with the topography of a surface on which the biofilm grows. The prevalence of *P. aeruginosa* occurs only when the culture is cultivated on a flat surface. During biofilm growth on undulating surfaces, *E. coli* bacilli are less likely to disperse. This phenomenon is related to the production of indole by *E. coli* cells, which reduces the dispersive response of these bacilli to signaling compounds secreted by *P. aeruginosa*. Moreover, indole was found to play a significant role in the survival of *E. coli* in a mixed biofilm by inhibiting supernatant obtained from *P. aeruginosa* biofilm culture strongly stimulated the growth of planktonic forms, however, they inhibited the formation of biofilm formed by *E. coli*. They also significantly reduced the survival rate of *E. coli*. Metabolites found in the supernatant obtained from *E. coli* biofilm culture did not affect either the growth of planktonic forms or biofilm mass formation by *P. aeruginosa.* However, they decreased the survival rate of *P. aeruginosa* in the biofilm. Castonguay et al. [47] conducted a study focusing on mixed biofilm formation by *E. coli* PHL565 and *Pseudomonas putida* MT2. It should be noted that these strains differed in terms of their adhesive capabilities. The former strain was non-adherent and thus incapable of forming a biofilm, while the latter one showed extraordinarily strong adhesive properties and formed a thin biofilm even on glass surfaces. Interestingly, in a mixed culture, *E. coli* PHL565 bacilli were able to adhere to surfaces and they formed a biofilm together with *P. putida*.

### 3.5. Formation of Biofilm Mass by Uropathogenic Rods in Mono-, Dual- and Triple-Species Biofilms Untreated with AA

The amount of biofilm mass created by individual species of bacteria allowed to differentiate them into weak, moderate, or strong biofilm producers. Depending on the optical density (OD) value, the strains were classified into one of 4 groups: OD_590_ ≤ 0.218—no biofilm, 0.218 < OD_590_ ≤ 0.436—weak biofilm, 0.436 < OD_590_ ≤ 0.872—moderate biofilm, and OD_590_ > 0.872—strong biofilm.

**Figure 5:**
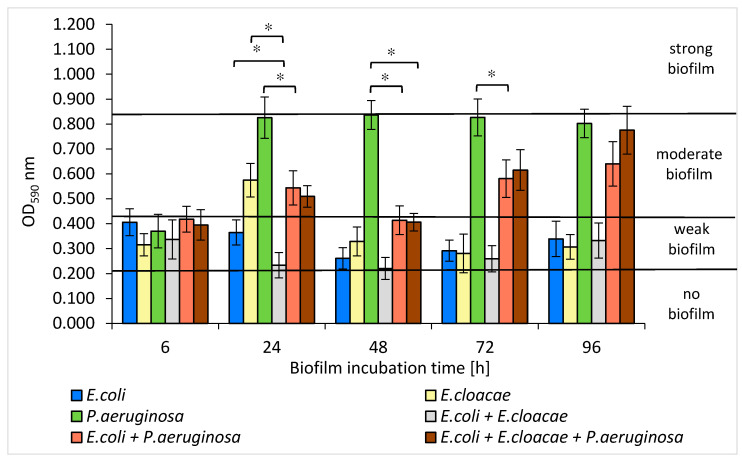
The biofilm mass production by bacteria living in mono-, dual-, and triple-species biofilm consortia. Statistically significant differences were noted with an asterisk (* *p* ≤ 0.05).

The *E. coli* strain formed a weak biofilm at all stages of development (Figure 5). The highest amount of biofilm was recorded in the 6 h culture (OD_590_ = 0.406), and the lowest in the 48 h one (OD_590_ = 0.261). The *E. cloacae* strain produced a weak biofilm (0.281 < OD_590_ ≤ 0.329) at all stages of its formation except for the 24 h culture in which the mean OD_590_ value was 0.574, indicating the average biofilm mass production. The *P. aeruginosa* strain was the best biofilm producer. It produced a moderate biofilm (0.802 < OD_590_ ≤ 0.836) at all stages of biofilm maturation, except for the 6 h culture (OD_590_ = 0.370).

As shown in Figure 5, *E. coli* and *E. cloacae* grown together in the 24, 48, and 72 h dual-species consortia produced less biofilm mass than when they were grown separately. However, statistical significance was only demonstrated in the 24 h culture (*p* ≤ 0.05).

An interesting result was obtained for the dual-species biofilm of *E. coli + P. aeruginosa* in comparison to the mono-species biofilm formed by *P. aeruginosa*. It is clear that the *E. coli* rods act antagonistically inhibiting EPS production by *P. aeruginosa*. The strongest inhibitory effect was observed especially in 24, 48, and 72 h cultures where the reduction in biofilm mass was 34%, 51%, and 30%, respectively (*p* ≤ 0.05) (Figure 5). Similar results were obtained for the triple-species biofilm of *E. coli + E. cloacae + P. aeruginosa*. The greatest reduction in biofilm mass synthesis (52%) was recorded in the 48 h culture in comparison to *P. aeruginosa* monoculture (*p* ≤ 0.05) (Figure 5).

According to our study, *E. coli* and *E. cloacae* formed weak single-species biofilms, while the *P. aeruginosa* strain had much stronger biofilm production. Moreover, Wang et al. [48] observed that the clinical strains of *P. aeruginosa* produced significantly greater biofilm mass than *E. coli*, *E. faecalis*, *S. aureus*, *K. pneumoniae*, *E. cloacae*, and *Klebsiella oxytoca*. The present study also found that the amount of produced biofilm mass was usually greater in single-species consortia compared to dual- and triple-species biofilms. The fact that the amount of biomass produced in mature mixed biofilms was smaller than the amount of biomass produced at the same stage of biofilm formation by each individual species provides evidence of antagonistic interactions between the analyzed bacteria. This was particularly evident in the dual-species biofilm, in which the amount of extracellular polymeric substance (EPS) produced by *P. aeruginosa* was decreased by the presence of *E. coli*. These findings contradict studies of other research groups [36,49,50,51]. Qian et al. [49] found that the amount of biofilm mass produced by single-species consortia of *E. coli* ATCC 25922 and *E. cloacae* ATCC 13047 was smaller than that in mixed biofilm. Kuznetsova et al. [36] also observed that the amount of biomass in dual-species biofilms composed of *E. coli* and *P. aeruginosa* was significantly greater compared to single-species biofilms. Similarly, Liu et al. [50] observed synergism during the formation of dual-species biofilms containing *Burkholderia caryophylli* and *E. coli* O157:H7, as well as *Ralstonia insidiosa* and *E. coli* O157:H7. Moreover, Culotti et al. [51] observed that the growth and synthesis of biofilm mass produced by *E. coli* DH5α were more intense when these bacteria grew together with *P. aeruginosa* PAO1. Interestingly, *P. aeruginosa* grew only in clusters, whereas *E. coli* occupied a much larger area, growing as a lawn. On the other hand, Machado et al. [33] found that the amount of biofilm mass formed by *P. aeruginosa* ATCC 10145 and *E. coli* K12MG1655 after the 6-day incubation was similar whether the bacteria grew in monocultures or formed a common consortium.

### 3.6. Metabolic Activity of Uropathogenic Rods in Mono-, Dual- and Triple-Species Biofilms Untreated with AA

**Figure 6:**
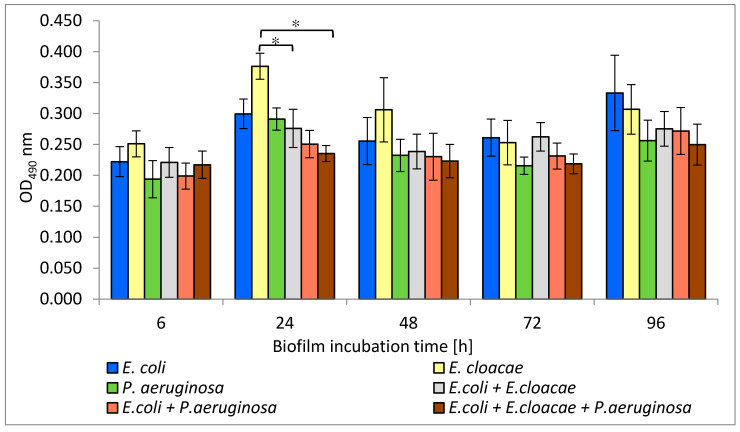
The metabolic activity of bacteria living in mono-, dual-, and triple-species biofilm consortia. Statistically significant differences were noted with an asterisk (* *p* ≤ 0.05).

The results presented in Figure 6 show that the highest metabolic activity was noticed in the 6, 24, and 48 h *E. cloacae* monocultures as well as in the 96 h monoculture of *E. coli*. The weaker metabolic activity was exhibited in the single-species *P. aeruginosa* cultures regardless of the stage of biofilm development. It was also observed that the simultaneous presence of *E. coli + E. cloacae* and *E. coli + E. cloacae + P. aeruginosa* resulted in a reduction in metabolic activity in 24 h cultures compared to the activity of *E. cloaceae* rods growing in a mono-species consortium (*p* ≤ 0.05). Therefore, it can be concluded that strains are mutually antagonistic. The metabolic activity of bacteria living in the dual-biofilm of *E. coli + P. aeruginosa* seems to be lower than the activity of *E. coli* growing in monoculture. In addition, the metabolic activity of bacteria growing in triple-species biofilms in 24, 48, and 96 h cultures was weaker than when these species lived in mono-species consortia, but the results have no statistical significance (*p* > 0.05).

Machado et al. [33] determined the metabolic activity of *P. aeruginosa* ATCC 10145 and *E. coli* K12MG1655 strains in 6-day mono-cultured biofilms. In a monoculture, *P. aeruginosa* had higher metabolic activity than *E. coli.* This result is contrary to the one obtained in our current study. According to our study, *P. aeruginosa* had weaker metabolic activity compared to *E. coli* and *E. cloacae*. However, this study and Machado et al.’s study [33] found that bacteria growing in mixed consortia had lower metabolic activity than single-species consortia.

Interestingly, the present study showed that the amount of formed biofilm mass does not correlate with the metabolic activity of the bacteria. *E. cloacae* and *E. coli* strains producing only a weak biofilm had higher metabolic activity than better biofilm-producing *P. aeruginosa.*

The present study also attempted to determine the effect of AA on survival, biofilm mass production, and metabolic activity of *E. coli*, *E. cloacae*, and *P. aeruginosa* growing in monocultures and in mixed biofilms. The obtained results indicate that AA has an antibacterial effect on bacteria growing in all tested biofilms.

### 3.7. The E. coli Cell Count in Mono-, Dual-, and Triple-Species Biofilms Treated with AA

**Figure 7:**
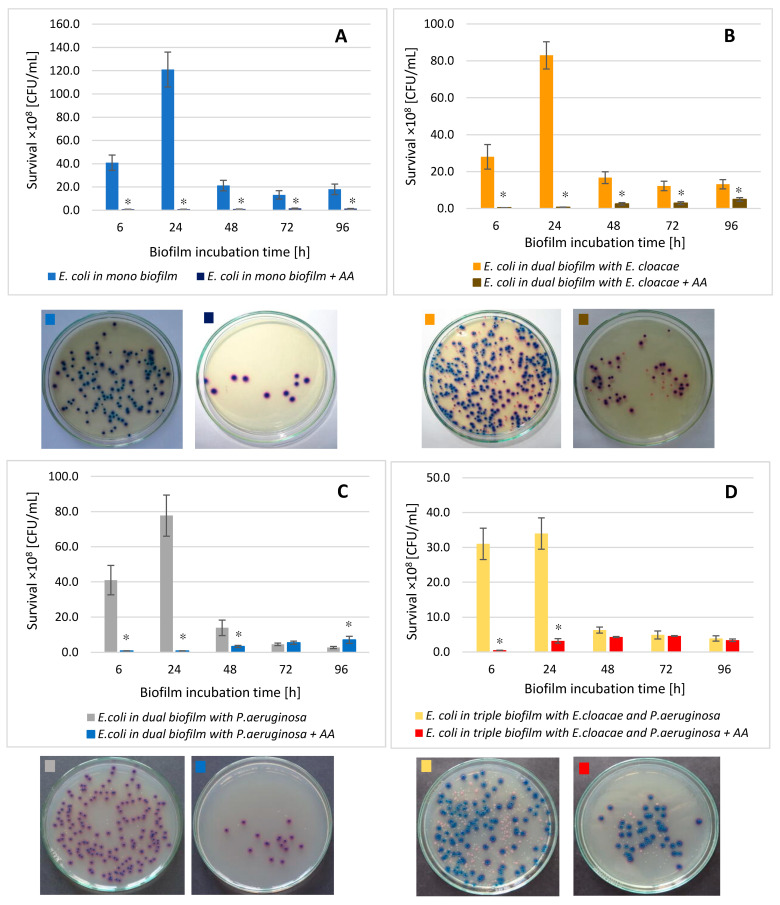
The survival of *E. coli* rods in mono- (**A**), dual- (**B**,**C**), and triple-species (**D**) biofilms treated with asiatic acid (AA) in comparison to untreated biofilms. Statistically significant differences were noted with an asterisk (* *p* ≤ 0.05). The photos show bacterial colonies grown on chromogenic coliform agar inoculated from 24-hour biofilm cultures.

In mono-species biofilm formed by *E. coli* (Figure 7A), regardless of its maturity stage, a statistically significant decrease in the number of viable bacteria under the influence of AA was noted in comparison with the control sample (*p* ≤ 0.05). The greatest reduction in the number of viable cells (4 log10) occurred after 24 h of incubation. The weakest effect of AA was recorded in the older 72 and 96 h biofilms (Figure 7A).

The data contained in Figure 7B,C show that this acid reduced the number of *E. coli* also in dual-species biofilms (*E. coli + E. cloacae* + AA and *E. coli + P. aeruginosa* + AA) as well as triple-species biofilm (*E. coli + E. cloacae + P. aeruginosa* + AA; Figure 7D) compared to the number of *E. coli* cells grown in the consortia untreated with AA.

Analyzing the effect of AA on the survival of bacteria in a dual-species biofilm (*E. coli + P. aeruginosa*; Figure 7C), it was noticed that the acid inhibited the growth of *E. coli* in the 6, 24, and 48 h biofilms and the reduction was 2.6 log10, 2.5 log10, and 0.6 log10, respectively (*p* ≤ 0.05). No inhibitory effect of AA on the survival of *E. coli* bacilli was observed only in the mature 72 and 96 h dual-species culture of *E. coli + P. aeruginosa +* AA.

**Figure 8:**
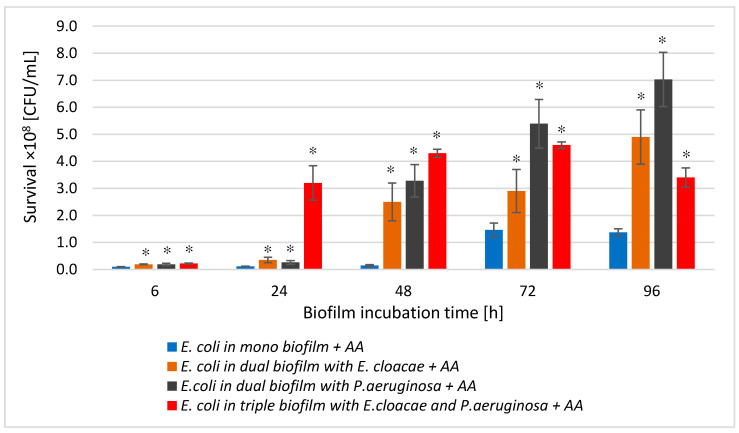
Comparison of the *E. coli* rods survival in mono-species biofilm with the survival of *E. coli* rods growing in dual- and triple-species consortia treated with asiatic acid (AA). Statistically significant differences were noted with an asterisk (* *p* ≤ 0.05).

Comparative analysis of *E. coli* survival in mono-species biofilm treated with AA with survival of these bacteria in dual-species (*E. coli + E. cloacae +* AA and *E. coli + P. aeruginosa +* AA) and triple-species (*E. coli + E. cloacae + P. aeruginosa +* AA) biofilms, also in the presence of the AA (Figure 8), showed that the acid was most effective against *E. coli* living in mono-species biofilm regardless of its stage of development (*p* ≤ 0.05). On the other hand, *E. coli* bacilli showed the lowest sensitivity to the action of AA, living in a triple-species consortium (*E. coli + E. cloacae + P. aeruginosa +* AA) in 6, 24, and 48 h cultures.

### 3.8. The E. cloacae Cell Count in Mono-, Dual-, and Triple-Species Biofilms Treated with AA

**Figure 9:**
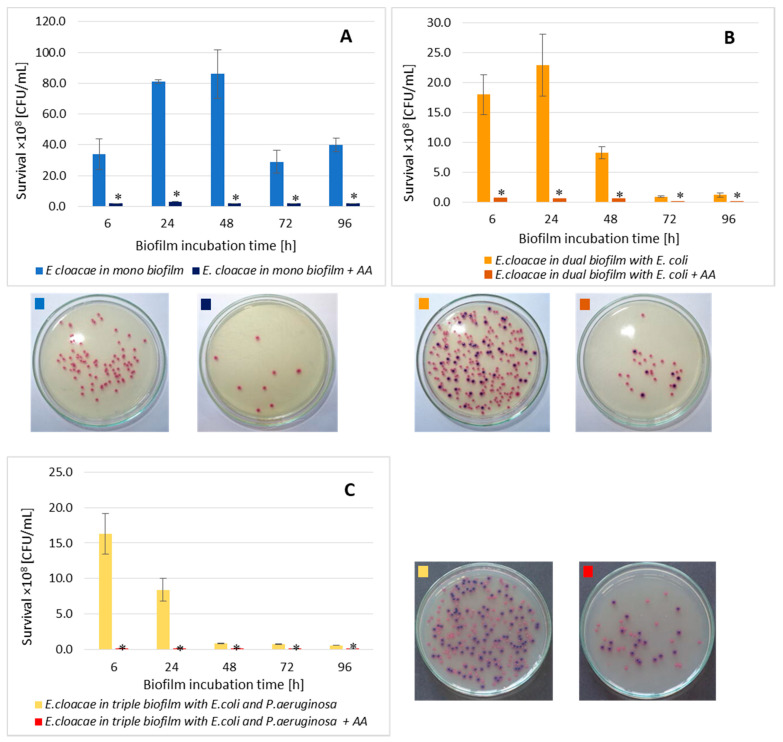
The survival of *E. cloacae* rods in mono- (**A**), dual- (**B**), and triple-species (**C**) biofilms treated with asiatic acid (AA) in comparison to untreated biofilms. Statistically significant differences were noted with an asterisk (* *p* ≤ 0.05). The photos show bacterial colonies grown on chromogenic coliform agar inoculated from 24-hour biofilm cultures.

As shown in Figure 9A, AA significantly reduced the *E. cloacae* cells count in mono-species biofilm at all stages of its development (*p* ≤ 0.05). The most effective antibacterial activity of the acid was recorded in the oldest 96 h biofilm (8.6 log10 reduction). The data in Figure 9 show that AA also significantly reduced the number of *E. cloacae* cells in both dual- (Figure 9B) and triple-species biofilm (Figure 9C) (*p* ≤ 0.05) at all stages of their development. 

**Figure 10:**
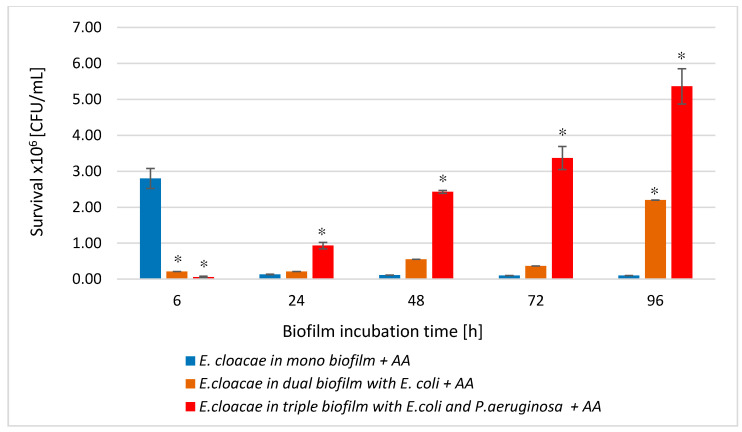
Comparison of the *E. cloacae* rods survival in mono-species biofilm with the survival of *E. cloacae* rods growing in dual- and triple-species consortia treated with asiatic acid (AA). Statistically significant differences were noted with an asterisk (* *p* ≤ 0.05).

Comparative analysis of the effect of AA on *E. cloacae* survival in mono-, dual-, and triple-species biofilms (Figure 10) showed that the acid had the weakest antibacterial effect on the *E. cloacae* grown a triple-species biofilm. The acid was most effective against *E. cloacae* living in mono-species biofilm regardless of its stage of development (*p* ≤ 0.05) with the exception of the young 6 h culture where *E. cloacae* rods grew much better than in dual- as well as triple-species consortia (*p* ≤ 0.05).

### 3.9. The P. aeruginosa Cell Count in Mono-, Dual-, and Triple-Species Biofilms Treated with AA

**Figure 11:**
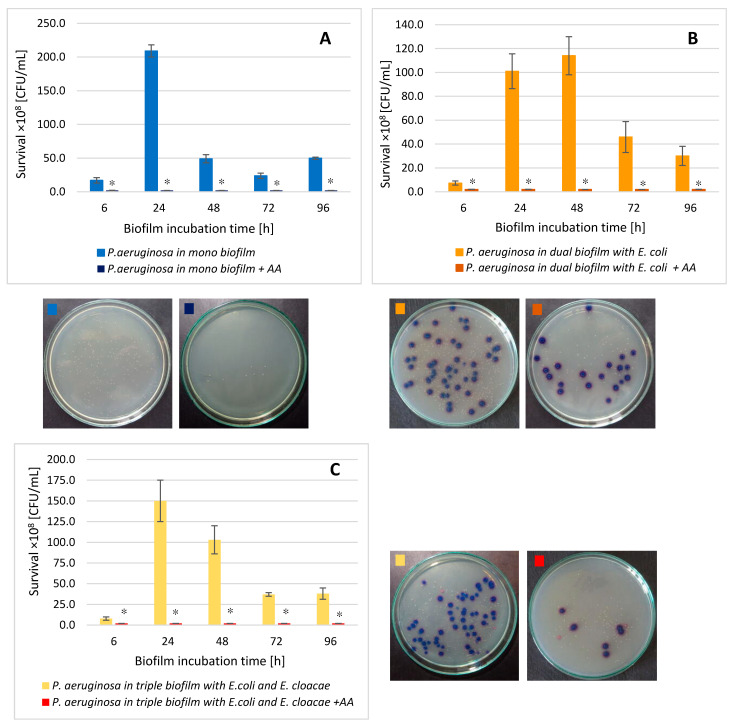
The survival of *P. aeruginosa* rods in mono- (**A**), dual- (**B**), and triple-species (**C**) biofilms treated with asiatic acid (AA) in comparison to untreated biofilms. Statistically significant differences were noted with an asterisk (* *p* ≤ 0.05). The photos show bacterial colonies grown on chromogenic coliform agar inoculated from 24-hour biofilm cultures.

The data presented in Figure 11 show that AA significantly reduced the number of viable *P. aeruginosa* cells in mono-, dual-, and triple-species biofilms compared to the untreated samples at all stages of their development (*p* ≤ 0.05). The greatest decrease in the *P. aeruginosa* cells count was recorded in 72 h mono- and triple-species biofilms (Figure 11C); reduction values were 3.2 log10 and 3.5 log10, respectively. In the dual-species biofilm (Figure 11B), the AA showed the best bactericidal activity on the *P. aeruginosa* rods grown in 48 and 72 h cultures with a growth reduction of 3.2 log10. 

**Figure 12:**
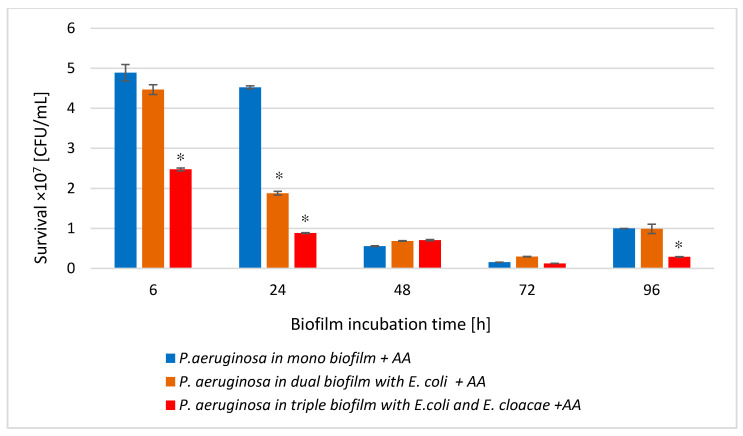
Comparison of the *P. aeruginosa* rods survival in mono-species biofilm with the survival of *E. cloacae* rods growing in dual- and triple-species consortia treated with asiatic acid (AA). Statistically significant differences were noted with an asterisk (* *p* ≤ 0.05).

Comparing the *P. aeruginosa* cells count in all AA-treated biofilms (Figure 12), it can be concluded that the acid exhibited the most effective antibacterial activity against *P. aeruginosa* rods growing in the 6, 24, and 96 h triple-biofilm cultures comparing them to mono-species consortia. This situation undoubtedly proves that the presence of additional species in the culture (*E. coli* and *E. cloacae*) strongly antagonizes the *P. aeruginosa* strain. As a result of these interactions, the *P. aeruginosa* strain becomes more susceptible to AA.

The reduction in the number of viable cells depended on the duration of AA and whether a bacterial strain grew in a single- or multi-species consortium. AA the most reduced bacterial counts in single-species biofilms while slightly less in dual- and triple-species biofilms (Figure 8 and Figure 10). AA also inhibited EPS synthesis in all tested biofilms. Under the influence of AA, there was also a decrease in the metabolic activity of bacilli living in single-species biofilms at all stages of their formation. Our previous studies determining the effect of AA on both Gram-negative [24], and Gram-positive [21], bacteria, as well as on the formation and eradication of single-species biofilms, showed its antibacterial activity. AA also supported the pharmacological effects of ciprofloxacin when removing mature *E. coli* biofilm from urinary catheters [24]. Garo et al. [23] investigated the influence of AA on single-species *P. aeruginosa* biofilm. AA showed antibiofilm activity. Furthermore, it acted synergistically with ciprofloxacin and tobramycin. Bacterial susceptibility to AA may be associated with membrane disintegration. This is confirmed by the results obtained by Liu et al. [19], in which they proved that AA caused membrane damage in both Gram-negative bacteria (*E. coli* O157:H7, *S. typhimurium*, *P. aeruginosa)* and Gram-positive ones (*L. monocytogenes*, *S. aureus*, *E. faecalis*, *B. cereus)*. Meanwhile, the results of two other research teams did not prove the antibiofilm effect of AA against Gram-positive bacteria [22,52]. Harnvoravongchai et al. [22] found that AA, even at concentrations several times greater than the MIC value, had no inhibitory effect on biofilm formation by clinical strains of *Clostridium difficile*. Moreover, Bharitkar et al. [52] observed that AA even at a high concentration of 1250 μg/mL did not inhibit the growth of *Lactobacillus acidophilus*.

The results obtained by other authors indicate that different antibacterial agents show better activity against single-species biofilms than multi-species biofilms. The results of this study, however, are difficult to discuss because they are novel. No research group has investigated the effects of AA on bacteria living in multispecies consortia so far. According to Schwering et al. [53], dual-species biofilms of *E. coli* and *E. cloacae* are up to 300 times more resistant to chlorine than single-species biofilms. Furthermore, Qian et al. [49] investigated the effect of luteolin on mixed biofilm formed by *E. coli* ATCC 25922 and *E. cloacae* ATCC 13047. Qian et al. [49] found that luteolin had a much weaker effect than when the bacteria were grown in monocultures. 

### 3.10. Formation of Biofilm Mass by Uropathogenic Rods in Mono-, Dual-, and Triple-Species Biofilms Treated with AA

**Figure 13:**
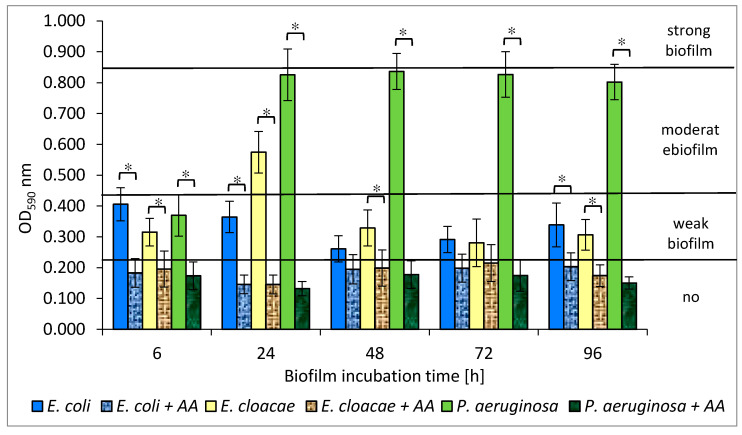
The influence of the asiatic acid (AA) on the biofilm-mass production by bacteria living in mono-species consortia. Statistically significant differences were noted with an asterisk (* *p* ≤ 0.05).

The data presented in Figure 13 shows that AA significantly inhibited the synthesis of biofilm formed by the rods growing in mono-species consortia (*p* ≤ 0.05). The exceptions were the 48 and 72 h *E. coli* cultures and a 72 h *E. cloacae* culture (*p* > 0.05). The most effective anti-biofilm activity of AA was observed in *P. aeruginosa* cultures. *P. aeruginosa* strain originally producing moderate biofilm (0.802 < OD_590_ ≤ 0.836) became a non-biofilm producer (OD_590_ ≤ 0.218).

**Figure 14:**
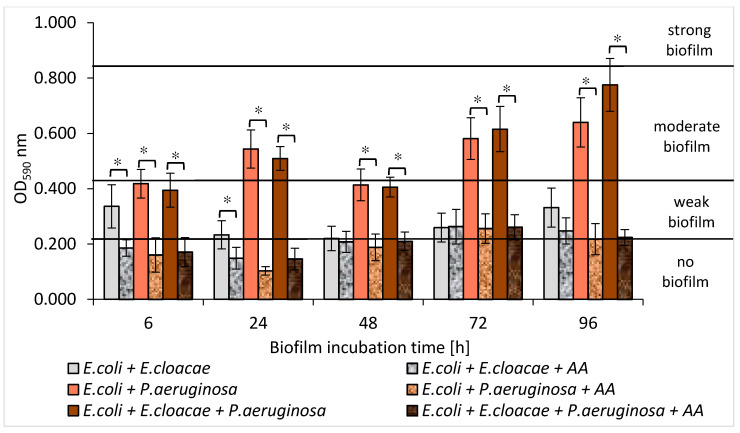
The influence of the asiatic acid (AA) on the biofilm-mass production by bacteria living in dual- and triple-species consortia. Statistically significant differences were noted with an asterisk (* *p* ≤ 0.05).

The results presented in Figure 14 show that AA reduced the amount of biofilm produced by bacteria growing in dual-species *E. coli + P. aeruginosa* and triple-species *E. coli + E. cloacae + P. aeruginosa* biofilms at all stages of their formation (*p* ≤ 0.05). However, in the dual-species *E. coli + E. cloacae* consortium, a significant reduction in biofilm mass under the influence of AA was recorded only in 6 and 24 h cultures (*p* ≤ 0.05). It is worth noting that complete inhibition of biofilm synthesis (OD_590_ ≤ 0.218) was noted in all 6 and 24 h dual- and triple-species consortia (Figure 14).

### 3.11. Metabolic Activity of Uropathogenic Rods in Mono-, Dual-, and Triple-Species Biofilms Treated with AA

**Figure 15:**
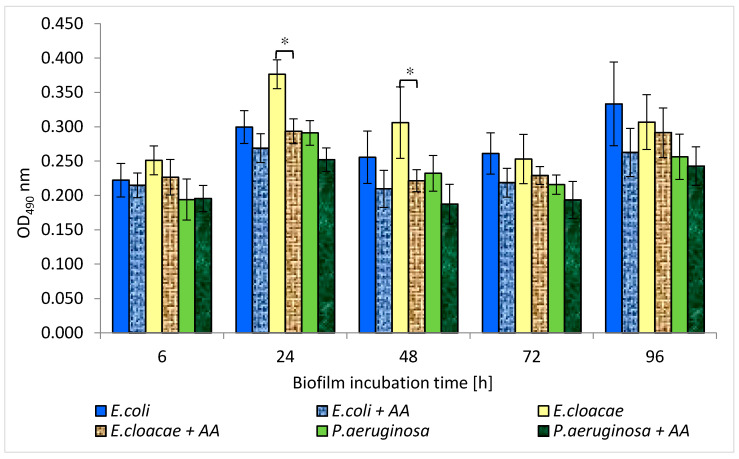
The influence of the asiatic acid (AA) on the metabolic activity of bacteria living in mono-species biofilms (* *p* ≤ 0.05).

**Figure 16:**
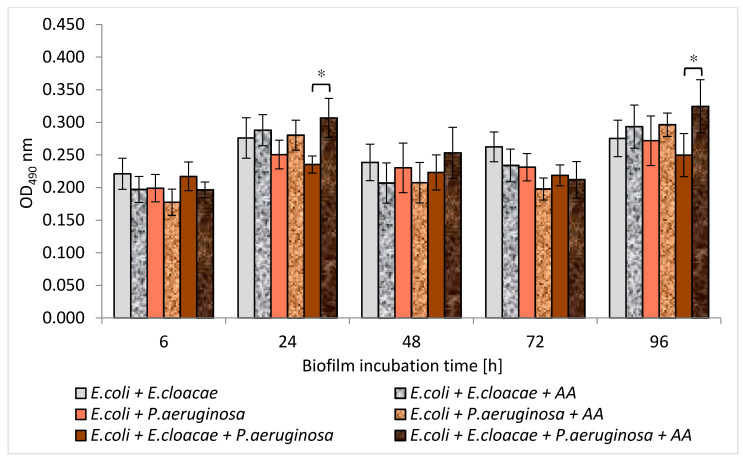
The influence of the asiatic acid (AA) on the metabolic activity of bacteria living in dual- and triple-species biofilms (* *p* ≤ 0.05).

The results in Figure 15 show that AA reduced the metabolic activity of rods growing in mono-species biofilms. However, statistically significant results were only reported for the 24 and 48 h *E. cloacae* cultures (*p* ≤ 0.05). It is worth emphasizing that AA did not reduce the metabolic activity of bacteria living in dual-species or triple-species biofilms (*p* > 0.05) (Figure 16). In contrast, the metabolic activity of bacteria growing in the triple-species consortia (24 and 96 h) significantly increased in the presence of AA (*p* ≤ 0.05). 

It is also difficult to discuss the results of our study regarding the effect of AA on the amount of formed biomass and the metabolic activity of bacteria living in biofilm consortia, due to the lack of literature data in this regard. Nostro et al. [54] described changes in the amount of biofilm and metabolic activity of *E. coli* and *S. aureus* that form a dual-species consortium under the influence of two plant-derived organic compounds—citronellol and eugenol. Citronellol had a better effect on single-species biofilms. The amount of biofilm mass was smaller and the bacterial metabolic activity was weaker compared to the dual-species consortium. In contrast, eugenol showed better antibiofilm activity on the dual-species consortium. 

### 3.12. Effect of AA on Bacterial Morphology

**Figure 17:**
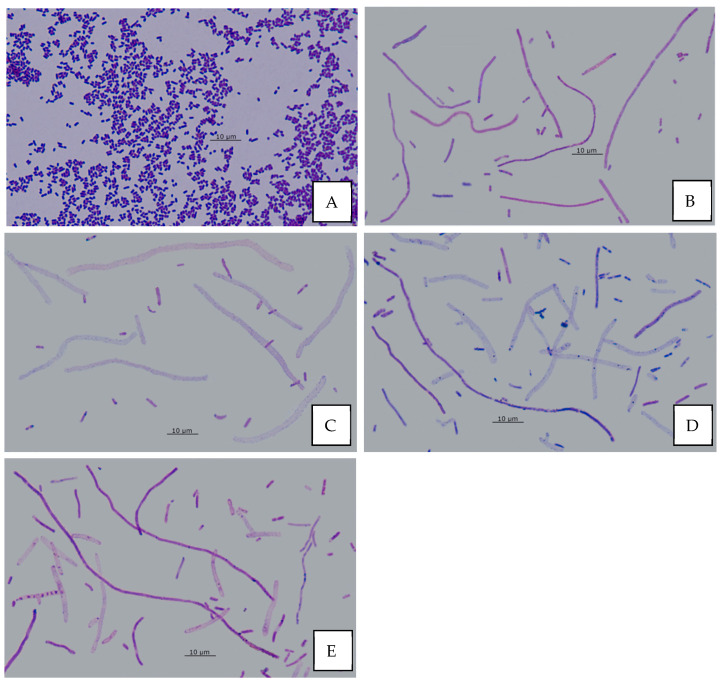
Morphological changes of bacterial cells under the treatment with asiatic acid (AA); (**A**)—untreated bacteria; (**B**–**E**)—samples treated with AA: (**B**)—long filaments in *E. coli + E. cloacae* biofilms (24–96 h); (**C**)—“ghost” cells in *E. coli* and *E. cloacae* biofilms (48–96 h); (**D**)—short filaments, long filaments and “ghost” cells in *E. coli + P. aeruginosa* biofilms (6–96 h); (**E**)—short filaments, long filaments and “ghost” cells in *E. coli + E. cloacae + P. aeruginosa* biofilms (24–96 h) (Nikon Eclipse 400; magnification, ×1000).

The normal cell length of the investigated strains ranges from 0.8 to 3.0 µm (Figure 17A). Various morphological changes were noted in AA-treated bacterial cells. In young 6 h monocultures of *E. coli* and *E. cloacae* short filaments (5–15 µm) were observed. In older biofilms (24–96 h) additionally, long filaments (>15 µm) have been found (Figure 17B). The so-called “ghost” cells (partially lacking cell wall) were present in single-species mature biofilms of *E. coli* (48–96 h) and *E. cloacae* (96 h) (Figure 17C). No morphological changes were observed in the biofilms formed by *P. aeruginosa*. Short and long filaments, as well as “ghost” cells, were observed in AA-treated dual-biofilm of *E. coli + P. aeruginosa*, regardless of its maturation stage (Figure 17D). In young 6 h dual-biofilms formed by *E. coli + E. cloacae* and triple-species biofilms (*E. coli + E. cloacae + P. aeruginosa*), only short filaments were found, while in older biofilms (24–96 h), additionally long filaments and “ghost” cells were observed (Figure 17E).

Our study showed that in the presence of AA, there were short filaments (5–15 μm) in young 6 h monoculture biofilms formed by *E. coli* and *E. cloacae,* and additionally long filaments (>15 µm) in older biofilms (24–96 h). “Ghost” cells were found in single-species mature biofilms of *E. coli* (48–96 h) and *E. cloacae* (96 h). On the other hand, no morphological changes were observed in mono-species biofilms formed by *P. aeruginosa*. In dual-species biofilms formed by AA-treated *E. coli* + *P. aeruginosa*, there were short and long filaments and “ghost” cells, regardless of the developmental stage of these biofilms (Figure 17D). On the other hand, in young 6 h biofilms formed by *E. coli* + *E. cloacae* there were only short filaments, while older biofilms (24–96 h) additionally contained long filaments and “ghost” cells. In a triple-species biofilm (*E. coli* + *E. cloacae + P. aeruginosa*) the same changes as in the dual-species biofilm formed by *E. coli* + *E. cloacae* were observed. In AA-treated biofilm cultures, there were changes in bacterial cellular morphology: short and long filaments and “ghost” cells. Similar changes were observed in microscopic images of *E. coli* cultures by Wojnicz et al. [17], who additionally found cells that were thickened, and they had intracellular edema. Wojnicz et al. [21] also conducted research concerning the effects of AA on biofilms formed by clinical *E. faecalis* strains. The cells of these cocci were larger in terms of diameter, and they formed irregular aggregates instead of chains.

It is known that morphological changes of bacterial cells are determined by different mechanisms. Cell filamentation may be caused by impaired synthesis of peptidoglycan that builds the cell wall or impaired formation of division septa. PBP3 (penicillin-binding protein 3) plays an important role in the formation of septa during bacterial cell division. Inhibition of this enzyme’s activity causes cell elongation without cell division. Βeta-lactam antibiotics, which have an affinity for PBP3, exhibit such division inhibitory activity. They cause filaments formation by *E. coli* and *P. aeruginosa* already at low antibiotic concentrations [55,56]. However, this is not the only mechanism that leads to the filamentation of bacterial cells. The filamentation of bacterial cells is also observed due to the inhibition of replication caused by DNA damage or dysfunction of the FtsZ protein, which is a key molecule for bacterial divisions. Delayed septum formation occurs due to inhibition of the FtsZ by the SulA protein. This inhibits the formation of the division ring and causes PBP3 inactivation. Cell filamentation is also observed under the influence of fluoroquinolones (e.g., ciprofloxacin) that contribute to blocking DNA replication by inhibiting gyrase activity [57,58]. It should be noted that both inhibitors of peptidoglycan synthesis and DNA synthesis may result in the creation of “ghost” cells in which partial lysis of the cell wall has occurred [59]. The mechanism of action of AA has yet to be thoroughly understood and described. In our current study, the observed morphological changes in *E. coli* and *E. cloacae* cells after exposure to AA confers the assumption that not only does it destroy the cell membrane integrity and cause its dysfunction, but it can also penetrate the bacterial cell and interact with DNA and/or proteins involved in the formation of the division septum. In this way, AA could affect the replication process and interfere with the cell division of the bacterial cell. Therefore, phenotypically altered bacteria may have a loss of ability to adhere to host cells, which consequently reduces their virulence.

## 4. Conclusions

The results of this study show significant effects of AA on the survival of bacterial cells, their morphology, ability to form single-, dual-, and triple-species biofilms, and metabolic activity of the cells living in them. However, there is a need for further research concerning AA as a documented bioactive substance, especially regarding its mechanism of action on bacterial cells. It is even more necessary to define principles of validating the antimicrobial activity of AA and principles of relating/converting the potency of its in vitro effect to its in vivo therapeutic effect. AA could then be used as both a complementary agent and adjunctive one for conventional antibiotic therapy applied for chronic UTIs. It could also be a preventive measure for people who are prone to recurrent UTIs. As AA worked best on young biofilms, the use of AA-containing formulations would be reasonable especially during the initial stages of infection.

## Figures and Tables

**Table 1:** MIC (minimum inhibitory concentration) and MBC (minimum bactericidal concentration) values of asiatic acid (AA) against bacterial strains.

		*E. coli* CFT073	*E. cloacae* BAA2468	*P. aeruginosa* ATCC 25000
**AA concentration (µg/mL)**	**MIC**	1536	1024	1536
	**MBC**	2048	1536	2048

## Data Availability

The data presented in this study are available on request from the corresponding author.

## References

[1] Lv J., Sharma A., Zhang T., Wu Y., Ding X. (2018). Pharmacological review on asiatic acid and its derivatives: A potential compound. SLAS Technol..

[2] Nagoor Meeran M.F., Goyal S.N., Suchal K., Sharma C., Patil C.R., Ojha S.K. (2018). Pharmacological properties, molecular mechanisms, and pharmaceutical development of asiatic acid: A pentacyclic triterpenoid of therapeutic promise. Front. Pharmacol..

[3] James J.T., Dubery I.A. (2009). Pentacyclic triterpenoids from the medicinal herb, *Centella asiatica* (L.) Urban. Molecules.

[4] Jäger S., Trojan H., Kopp T., Laszczyk M.N., Scheffler A. (2009). Pentacyclic triterpene distribution in various plants—Rich sources for a new group of multi-potent plant extracts. Molecules.

[5] Karłowicz-Bodalska K., Han S., Han T., Miranowicz M., Bodalska A. (2013). *Centella asiatica* (L.) Urban, syn. Hydrocotyle asiatica L.– Asian pennywort—A famous medicinal plant of the Far East. Post. Fitoter..

[6] Sun B., Wu L., Wu Y., Zhang C., Qin L., Hayashi M., Kudo M., Gao M., Liu T. (2020). Therapeutic potential of *Centella asiatica* and its triterpenes: A review. Front. Pharmacol..

[7] Thanusha A.V., Dinda A.K., Koul V. (2018). Evaluation of nano hydrogel composite based on gelatin/HA/CS suffused with asiatic acid/ZnO and CuO nanoparticles for second degree burns. Mater. Sci. Eng. C Mater. Biol. Appl..

[8] Yuyun X., Xi C., Qing Y., Lin X., Ke R., Bingwei S. (2018). Asiatic acid attenuates lipopolysaccharide-induced injury by suppressing activation of the Notch signaling pathway. Oncotarget.

[9] Han Y., Jiang Y., Li Y., Wang M., Fan T., Liu M., Ke Q., Xu H., Yi Z. (2019). An aligned porous electrospun fibrous scaffold with embedded asiatic acid for accelerating diabetic wound healing. J. Mater. Chem. B..

[10] Liew K.Y., Hafiz M.F., Chong Y.J., Harith H.H., Israf D.A., Tham C.L. (2020). A review of malaysian herbal plants and their active constituents with potential therapeutic applications in sepsis. Evid. Based Complement. Alternat. Med..

[11] Bylka W., Znajdek-Awizeń P., Studzińska-Sroka E., Dańczak-Pazdrowska A., Brzezińska M. (2014). *Centella asiatica* in dermatology: An overview. Phytother Res..

[12] Chandrika U.G., Prasad Kumarab P.A. (2015). Gotu Kola (*Centella asiatica*): Nutritional properties and plausible health benefits. Adv. Food Nutr. Res..

[13] Djoukeng J.D., Abou-Mansour E., Tabacchi R., Tapondjou A.L., Bouda H., Lontsi D. (2005). Antibacterial triterpenes from *Syzygium guineense* (Myrtaceae). J. Ethnopharmacol..

[14] Acebey-Castellon I.L., Voutquenne-Nazabadioko L., Doan Thi Mai H., Roseau N., Bouthagane N., Muhammad D., Le Magrex Debar E., Gangloff S.C., Litaudon M., Sevenet T. (2011). Triterpenoid saponins from *Symplocos lancifolia*. J. Nat. Prod..

[15] Norzaharaini M.G., Wan Norshazwani W.S., Hasmah A., Nor Izani N.J., Rapeah S. (2011). Preliminary study on antimicrobial activities of asiaticoside and asiatic acid against selected gram positive and gram-negative bacteria. Health Environ. J..

[16] Wong K.C., Hag Ali D.M., Boey P.L. (2012). Chemical constituents and antibacterial activity of *Melastoma malabathricum* L.. Nat. Prod. Res..

[17] Wojnicz D., Kicia M., Tichaczek-Goska D. (2013). Effect of asiatic and ursolic acids on morphology, hydrophobicity and adhesion of UPECs to uroepithelial cells. Folia Microbiol..

[18] Wojnicz D., Tichaczek-Goska D., Kicia M. (2013). Effect of asiatic and ursolic acids on growth and virulence factors of uropathogenic *Escherichia coli* strains. Turk. J. Biol..

[19] Liu W.H., Liu T.C., Mong M.C. (2015). Antibacterial effects and action modes of asiatic acid. Biomedicine.

[20] Ashella S., Fleming A.T. (2016). Antimicrobial activity of asiatic acid against bacteria and fungi. IJSR Online.

[21] Wojnicz D., Tichaczek-Goska D., Korzekwa K., Kicia M., Hendrich A. (2017). Anti-enterococcal activities of pentacyclic triterpenes. Adv. Clin. Exp. Med..

[22] Harnvoravongchai P., Chankhamhaengdecha S., Ounjai P., Singhakaew S., Boonthaworn K., Janvilisri T. (2018). Antimicrobial effect of asiatic acid against *Clostridium difficile* is associated with disruption of membrane permeability. Front. Microbiol..

[23] Garo E., Eldridge G.R., Goering M.G., DeLancey Pulcini E., Hamilton M.A., Costerton J.W., James G.A. (2007). Asiatic acid and corosolic acid enhance the susceptibility of *Pseudomonas aeruginosa* biofilms to tobramycin. Antimicrob. Agents Chemother..

[24] Wojnicz D., Tichaczek-Goska D., Kicia M. (2015). Pentacyclic triterpenes combined with ciprofloxacin help to eradicate the biofilm formed in vitro by *Escherichia coli*. Indian J. Med. Res..

[25] CLSI (2018). Methods for Dilution Antimicrobial Susceptibility Test for Bacteria That Grow Aerobically.

[26] Di Bonaventura G., Spedicato I., D’Antonio D., Robuffo I., Piccolomini R. (2004). Biofilm formation by *Stenotrophomonas maltophilia* modulation by quinolones, trimethoprim, sulfamethoxazole, and ceftazidime. Antimicrob. Agents Chemother..

[27] O’Toole G.A., Kolter R. (1998). Flagellar and twitching motility are necessary for *Pseudomonas aeruginosa* biofilm development. Mol. Microbiol..

[28] Stepanović S., Vuković D., Hola V., Di Bonaventura G., Djukić S., Cirković I., Ruzicka F. (2007). Quantification of biofilm in microtiter plates: Overview of testing conditions and practical recommendations for assessment of biofilm production by staphylococci. APMIS.

[29] Singh A.K., Prakash P., Achra A., Singh G.P., Das A., Singh R.K. (2017). Standardization and classification of in vitro biofilm formation by clinical isolates of *Staphylococcus aureus*. J. Global. Infect. Dis..

[30] Kim S., Kim M.J., Kang H.Y., Seol S.Y., Cho D.T., Kim J. (2010). A simple colorimetric method for testing antimicrobial susceptibility of biofilmed bacteria. J. Microbiol..

[31] Haney E.F., Trimble M.J., Cheng J.T., Vallé Q., Hancock R.E.W. (2018). Critical assessment of methods to quantify biofilm growth and evaluate antibiofilm activity of host defence peptides. Biomolecules.

[32] Taemchuay D., Rukkwamsuk T., Sakpuaram T., Ruangwises N. (2009). Antibacterial activity of crude extracts of *Centella asiatica* against *Staphylococcus aureus* in bovine mastitis. Kasetsart Vet..

[33] Machado I., Lopes S.P., Sousa A.M., Pereira M.O. (2012). Adaptive response of single and binary *Pseudomonas aeruginosa* and *Escherichia coli* biofilms to benzalkonium chloride. J. Basic Microbiol..

[34] Vanysacker L., Denis C., Declerck P., Piasecka A., Vankelecom I.F. (2013). Microbial adhesion and biofilm formation on microfiltration membranes: A detailed characterization using model organisms with increasing complexity. Biomed. Res. Int..

[35] Cerqueira L., Oliveira J.A., Nicolau A., Azevedo N.F., Vieira M.J. (2013). Biofilm formation with mixed cultures of *Pseudomonas aeruginosa/Escherichia coli* on silicone using artificial urine to mimic urinary catheters. Biofouling.

[36] Kuznetsova M.V., Maslennikova I.L., Karpunina T.I., Nesterova L.Y., Demakov V.A. (2013). Interactions of *Pseudomonas aeruginosa* in predominant biofilm or planktonic forms of existence in mixed culture with *Escherichia coli* in vitro. Can. J. Microbiol..

[37] Oliveira A., Sousa J.C., Silva A.C., Melo L.D.R., Sillankorva S. (2018). Chestnut honey and bacteriophage application to control *Pseudomonas aeruginosa* and *Escherichia coli* biofilms: Evaluation in an ex vivo wound model. Front. Microbiol..

[38] Solis-Velazquez O.A., Gutiérrez-Lomelí M., Guerreo-Medina P.J., Rosas-García M.L., Iñiguez-Moreno M., Avila-Novoa M.G. (2020). Nosocomial pathogen biofilms on biomaterials: Different growth medium conditions and components of biofilms produced in vitro. J. Microbiol. Immunol. Infect..

[39] Mirani Z.A., Fatima A., Urooj S., Aziz M., Khan M.N., Abbas T. (2018). Relationship of cell surface hydrophobicity with biofilm formation and growth rate: A study on *Pseudomonas aeruginosa, Staphylococcus aureus*, and *Escherichia coli*. Iran J. Basic Med. Sci..

[40] Davies D.G., Marques C.N.H. (2009). A fatty acid messenger is responsible for inducing dispersion in microbial biofilms. J. Bacteriol..

[41] Rahmani-Badi A., Sepehr S., Mohammadi P., Soudi M.R., Babaie-Naiej H., Fallahi H. (2014). A combination of cis-2-decenoic acid and antibiotics eradicates pre-established catheter-associated biofilms. J. Med. Microbiol..

[42] Tiwari R., Karthik K., Rana R., Malik Y.S., Dhama K., Joshi S.K. (2016). Quorum sensing inhibitors/antagonists countering food spoilage bacteria-need molecular and pharmaceutical intervention for protecting current issues of food safety. Int. J. Pharmacol..

[43] Cao T., Morales-Soto N., Jia J., Baig N.F., Dunham S.J.B., Ellis J., Sweedler J.V., Shrout J.D., Bohn P.W. (2019). Spatiotemporal dynamics of molecular messaging in bacterial co-cultures studied by multimodal chemical imaging. Proc. SPIE Int. Soc. Opt. Eng..

[44] Bhattacharjee A., Khan M., Kleiman M., Hochbaum A.I. (2017). Effects of growth surface topography on bacterial signaling in coculture biofilms. ACS Appl. Mater. Interfaces.

[45] Chu W., Zere T.R., Weber M.M., Wood T.K., Whiteley M., Hidalgo-Romano B., Valenzuela E., McLean R.J. (2012). Indole production promotes *Escherichia coli* mixed-culture growth with *Pseudomonas aeruginosa* by inhibiting quorum signaling. Appl. Environ. Microbiol..

[46] Lopes S.P., Machado I., Pereira M.O. (2011). Role of planktonic and sessile extracellular metabolic byproducts on *Pseudomonas aeruginosa* and *Escherichia coli* intra and interspecies relationships. J. Ind. Microbiol. Biotechnol..

[47] Castonguay M.H., van der Schaaf S., Koester W., Krooneman J., van der Meer W., Harmsen H., Landini P. (2006). Biofilm formation by *Escherichia coli* is stimulated by synergistic interactions and co-adhesion mechanisms with adherence-proficient bacteria. Res. Microbiol..

[48] Wang X., Lünsdorf H., Ehrén I., Brauner A., Römling U. (2010). Characteristics of biofilms from urinary tract catheters and presence of biofilm-related components in *Escherichia coli*. Curr. Microbiol..

[49] Qian W., Fu Y., Liu M., Zhang J., Wang W., Li J., Zeng Q., Wang T., Li Y. (2021). Mechanisms of action of luteolin against single- and dual-species of *Escherichia coli* and *Enterobacter cloacae* and its antibiofilm activities. Appl. Biochem. Biotechnol..

[50] Liu N.T., Nou X., Lefcourt A.M., Shelton D.R., Lo Y.M. (2014). Dual-species biofilm formation by *Escherichia coli* O157:H7 and environmental bacteria isolated from fresh-cut processing facilities. Int. J. Food Microbiol..

[51] Culotti A., Packman A.I. (2014). *Pseudomonas aeruginosa* promotes *Escherichia coli* biofilm formation in nutrient-limited medium. PLoS ONE.

[52] Bharitkar Y.P., Banerjee M., Kumar S., Paira R., Meda R., Kuotsu K., Mondal N.B. (2013). Search for a potent microbicidal spermicide from the isolates of *Shorea robusta* resin. Contraception.

[53] Schwering M., Song J., Louie M., Turner R.J., Ceri H. (2013). Multi-species biofilms defined from drinking water microorganisms provide increased protection against chlorine disinfection. Biofouling.

[54] Nostro A., Scaffaro R., D’Arrigo M., Botta L., Filocamo A., Marino A., Bisignano G. (2013). Development and characterization of essential oil component-based polymer films: A potential approach to reduce bacterial biofilm. Appl. Microbiol. Biotechnol..

[55] Kong K.F., Schneper L., Mathee K. (2010). Beta-lactam antibiotics: From antibiosis to resistance and bacteriology. APMIS.

[56] El-Hajj Z.W., Newman E.B. (2015). How much territory can a single *E. coli* cell control?. Front. Microbiol..

[57] Justice S.S., Hunstad D.A., Seed P.C., Hultgren S.J. (2006). Filamentation by *Escherichia coli* subverts innate defenses during urinary tract infection. Proc. Natl. Acad. Sci. USA.

[58] Cheng G., Hao H., Dai M., Liu Z., Yuan Z. (2013). Antibacterial action of quinolones: From target to network. Eur. J. Med. Chem..

[59] Cushnie T.P., O’Driscoll N.H., Lamb A.J. (2016). Morphological and ultrastructural changes in bacterial cells as an indicator of antibacterial mechanism of action. Cell. Mol. Life Sci..

